# Exposure to COVID-19 Infection and Mortality Rates Among People With Disabilities in South Korea

**DOI:** 10.34172/ijhpm.2022.6996

**Published:** 2022-06-27

**Authors:** Woo-Hwi Jeon, In-Hwan Oh, Jeong-Yeon Seon, Jin-Nam Kim, So-Youn Park

**Affiliations:** ^1^Department of Preventive Medicine, School of Medicine, Kyung Hee University, Seoul, South Korea; ^2^Health Insurance Research Institute, National Health Insurance Service, Wonju, South Korea.; ^3^Department of Health Services Management, School of Management, Kyung Hee University, Seoul, South Korea.; ^4^Department of Medical Education and Medical Humanities, School of Medicine, Kyung Hee University, Seoul, South Korea.

**Keywords:** COVID-19, People With Disabilities, Types of Disabilities, Infection, Mortality, South Korea

## Abstract

**Background:** With the delayed eradication of coronavirus disease 2019 (COVID-19), people with disabilities, a socially vulnerable class of individuals, face aggravated hardships caused by a pause in support services and lack of care due to stricter social distancing policies combined with the challenges of their disabilities. Given this background, we aim to investigate COVID-19 infection and mortality rates among people with disabilities, who face heightened physical and mental health threats amidst the COVID-19 pandemic.

**Methods:** Gender, age, health insurance premiums, the Charlson Comorbidity Index (CCI), the severity of the disability, and the type of disability were compared among people with disabilities who had been infected with or died from COVID-19 using the nationally representative National Health Insurance Service (NHIS)-COVID-19 database (DB).

**Results:** We found that the COVID-19 infection rate was higher among those with low income, those with severe disability, and those with "other" disabilities (ie, speech disabilities, hepatic dysfunction, respiratory dysfunction, facial disfigurement, intestinal fistular/urinary disability, epilepsy, intellectual disability, autistic disorder, and mental disorders). The mortality rate was markedly higher (ie, 15.90 times higher, odds ratio [OR]: 15.90, 95% confidence interval [CI]: 6.16 - 41.06) among people aged 80 years or older as compared with those aged 60 years or younger. The odds for mortality were 2.49 times higher (OR: 2.49, 95% CI: 1.33 - 4.64) among people with severe disabilities as compared with mild disabilities.

**Conclusion:** Among people with disabilities, we found that COVID-19 infection rates differed according to income level, severity of the disability, and disability type, while the COVID-19 mortality rates differed according to age and severity of the disability.

## Background

**Key Messages**

** Implications for policy makers**

- The nationally representative National Health Insurance Service-coronavirus disease 2019 (NHIS-COVID-19) database (DB) was used for the study, which provides accurate and reliable information about the current state of COVID-19 among people with disabilities in Korea.
- As existing studies on COVID-19 infection and mortality primarily compare between those with and without disabilities, the greatest implication of this study is that it shed light on the risk factors for COVID-19 infection and mortality within the disabled population based on various individual characteristics.
- As supported by this study, appropriate infection containment measures and response manuals tailored to different ages, income levels, and severities and types of disabilities should be developed to prepare for potential infectious outbreaks in the future.

** Implications for the public**

Most people with disabilities have challenges in carrying on with their daily lives without the assistance of others provided through welfare and caregiving services. The findings of this study showed that the degree and types of assistance needed vary according to the specific characteristics of people with disabilities. In particular, people with severe disabilities and elderly people with disabilities need careful attention and management. Hence, family caregivers or other care providers should have an accurate understanding of the level of challenges and risks in each individual in order to provide tailored services and take appropriate precautions so as to contain the spread of the infection to vulnerable people with disabilities.

Coronavirus disease 2019 (COVID-19), which is near the end of its second year (as of November 2021; the first index case was identified in December 2019), shows no signs of wavering, with a cumulative total of 250 154 972 confirmed cases and 5 054 267 deaths occurring worldwide.^1^ The changes in daily life provoked by the persistent pandemic pose meaningful health threats to many. Reduced physical activity, a result of social distancing, has an adverse impact on physical as well as mental health, leading to problems such as anxiety, depression, and high stress.^2^

 These times are toughest on those with disabilities, a socially vulnerable group of individuals. With the prolonged pandemic and the enforcement of stricter social distancing policies, people with disabilities suffer from an array of problems throughout their lives, adding to their existing challenges from their disabilities, their difficulty in utilizing services from social support systems, and their lack of family support and care.^3,4^ Further, institutionalized people with disabilities are exposed to high risks of infection on the pretext of enforced isolation as a cohort and are suffering from extreme loneliness and depression; likewise,people with disabilities are battling COVID-19 in an environment that threatens their health to a greater degree than within the general population.^5^

 COVID-19 also poses other problems for people with disabilities.^6^ The disabled population is highly vulnerable to the exposure of the infectious disease itself.^7^ A recent study reported that disability, along with older age and pre-existing conditions, is also a risk factor for severe COVID-19 morbidity and mortality. A British study that analyzed the prognoses of COVID-19 patients according to their disability status from January 2020 to November 2020 reported that COVID-19 mortality was 3.5 times higher among patients with severe disabilities and 1.9 times higher among those with mild disabilities as compared to their non-disabled counterparts.^8^ A study conducted on people with and without disabilities in New York (United States) also reported similar results. In the said study, the COVID-19 fatality rate was higher (15%) among people with physical disabilities in New York as compared with their non-disabled counterparts (7.9%), with a markedly higher number of deaths per 100 000 population (1175 deaths) in the former group as compared to the latter group (151 deaths).^9^

 Results indicating that those with disabilities are more vulnerable to the exposure of COVID-19 are also consistently reported in Korea. According to a recent study on 129 120 people who were tested for COVID-19 between January and May 2020 (including 7261 people with disabilities), the odds for a positive test result was 1.36 times higher and the risk for poor clinical outcomes due to COVID-19 was 1.43 times higher among people with disabilities as compared to the non-disabled population.^10^ Additionally, a government report showed that 1562 out of 39 432 (4%) COVID-19-confirmed patients as of December 9, 2020 were people with disabilities, and 117 out of 556 deaths (21%) were among people with disabilities. The odds for death from COVID-19 infection were 6.5 times higher among people with disabilities as compared with their non-disabled counterparts; the COVID-19 fatality rate was 7.49% (117 out of 1562) among people with disabilities as compared to 1.15% (439 out of 37 870) in the non-disabled population.^11^

 Despite reports of the challenges and negative outcomes of COVID-19 among people with disabilities, national-level studies as well as surveys of COVID-19-related status among people with disabilities are lacking in Korea. Additionally, institutional systems for COVID-19 response among people with disabilities are yet to be established. Thus, this study aimed to analyze the association between disability and socioeconomic characteristics of individuals with disabilities and exposure to COVID-19 infection and mortality rates with the ultimate goal of raising public awareness regarding people with disabilities (who are especially vulnerable to COVID-19) and preventing critical health hazards in this population.

## Materials and Methods

###  Data Collection and Study Participants

 In the current study, we used the National Health Insurance Service (NHIS)-COVID database (DB), a nationally representative DB, to compare the characteristics of people with disabilities who contracted COVID-19 infection as well as those who died from COVID-19 to their non-infected counterparts. The NHIS provides the NHIS-COVID-19 DB in order to support research attempting to discover evidence for treating COVID-19 patients as well as informing relevant policy-making. The NHIS-COVID-19 DB provides information about national health insurance (NHI) eligibility, premiums, health examination results, and medical service details for COVID-19 patients and healthy controls.^12^ The DB contains information on all medical services provided to COVID-19 patients and controls from May 1, 2015 to July 2020. The control group was 15 times larger than the COVID-19 patient group in the current study; the participants were stratified from among people without a COVID-19 testing history. The stratification variables were gender (male, female) and age (0–9, 10–19, 20–29, 30–39, 40–49, 50–59, 60–69, 70–79, and ≥80 years); participants were randomly sampled from each stratum.^12^ In this study, a person with a disability was defined strictly as a registered person with a disability per the Act on Welfare of Persons with Disabilities; 7621 people with disabilities included in the NHIS-COVID-19 DB were analyzed (Figure).

**Figure:**
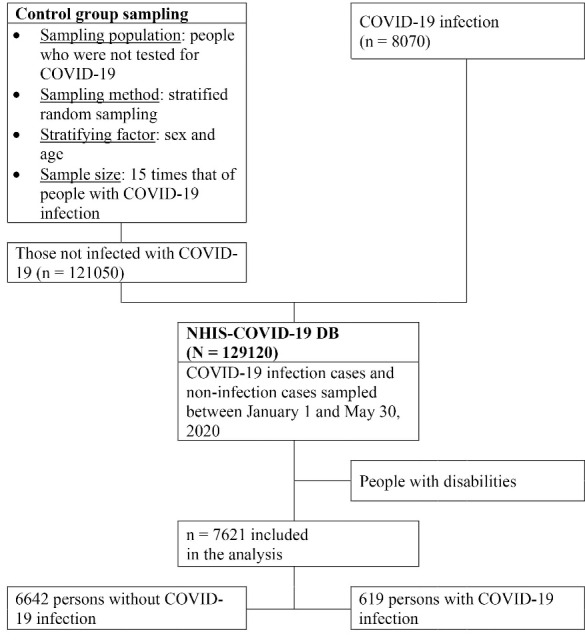
Flow Chart for Enrolling Study Participants. Abbreviations: COVID-19, coronavirus disease 2019, DB, database.

###  Key Variables

 The variables for the analysis included gender, age, health insurance premiums, the Charlson Comorbidity Index (CCI), the severity of the disability, and disability type. Age was classified into 10-year units (0–9, 10–19, 20–29, 30–39, 40–49, 50–59, 60–69, 70–79, 80+). We used health insurance premiums as a variable representing economic status, a major study parameter. In Korea, health insurance premiums vary depending on income level. The low-income class qualifies for Medical Aid with no premium; in the current study, NHI premiums were divided into five quantiles. The CCI indicates the severity of a pre-existing disease; in this study, scores were weighted by the updated weighting system proposed by Quan (based on 2019 medical service records).^13^ Disability severity and type were also divided into different groups. Severity was defined based on the disability grade, confirmed during the disability registration process; grades 1–3 were considered severe and grades 4–6 were considered mild. At the time of initial registration by an individual with disability, disabilities are classified into 15 types (physical disabilities such as limb amputation or deformity, brain lesions, visual disabilities, hearing disabilities, speech disabilities, kidney dysfunction, respiratory dysfunction, hepatic dysfunction, facial disfiguration, intestinal fistular/urinary disabilities, epilepsy, intellectual disabilities, autistic disorder, and mental disorders). However, for the purpose of de-identification, the NHIS-COVID-19 DB classifies disabilities into only five types: physical disabilities, brain lesions, visual disabilities, hearing disabilities, and other disability categories.

###  Statistical Analysis

 First, the medical and demographic characteristics associated with COVID-19 infection and death among people with disabilities were compared via the chi-square test. To follow, the association between economic status and participant characteristics and exposure risk to COVID-19 infection and mortality risk were analyzed using logistic regression. All logistic regression models were adjusted for gender, age, and CCI. Health insurance premiums as well as severity and type of disability were entered as interest variables, with COVID-19 infection as the outcome variable. Additionally, the logistic regression model with COVID-19 mortality as the outcome variable only included COVID-19 confirmed individuals with disabilities; health insurance premiums and severity and type of disability were entered as explanatory variables. Statistical analyses were performed using the SAS 9.4 software (Cary, NC, USA), and statistical significance was set at a *P* value of <.05.

## Results

###  Patient Characteristics

 Differences in COVID-19 infection and mortality rates according to gender, age, health insurance premiums, CCI, disability severity, and disability type were analyzed (Table 1).

**Table 1:** Baseline Characteristics Among People With Disabilities

	**People With Disabilities (n=7621)**					**People With Disabilities With COVID-19 (n=619**)				
	**Non-COVID-19 (n = 6642)**		**COVID-19 (n = 619)**		***P *** **Value**	**Survival (n = 546)**		**Death (n = 73)**		***P *** **Value**
	**n**	**%**	**n**	**%**		**n**	**%**	**n**	**%**	
Gender					.12					.41
Male	3355	50.5	333	53.8		297	54.4	36	49.3	
Female	3287	49.5	286	46.2		249	45.6	37	50.7	
Age (y)					<.01					<.01
0-9	14	0.2	0	0.0		0	0.0	0	0.0	
10-19	44	0.7	8	1.3		8	1.5	0	0.0	
20-29	435	6.5	23	3.7		23	4.2	0	0.0	
30-39	221	3.3	20	3.2		20	3.7	0	0.0	
40-49	405	6.1	50	8.1		49	9.0	1	1.4	
50-59	1112	16.7	127	20.5		117	21.4	10	13.7	
60-69	1503	22.6	167	27.0		155	28.4	12	16.4	
70-79	1441	21.7	118	19.1		104	19.0	14	19.2	
80+	1467	22.1	106	17.1		70	12.8	36	49.3	
Health insurance premium					<.01					.68
Medical aid	1290	19.4	246	39.7		220	40.3	26	35.6	
1st quintile (lowest)	1118	16.8	106	17.1		94	17.2	12	16.4	
2nd quintile	633	9.5	36	5.8		31	5.7	5	6.8	
3rd quintile	868	13.1	51	8.2		46	8.4	5	6.8	
4th quintile	1038	15.6	70	11.3		63	11.5	7	9.6	
5th quintile (highest)	1695	25.5	110	17.8		92	16.8	18	24.7	
CCI score					.13					<.01
0	3739	56.3	327	52.8		304	55.7	23	31.5	
1	1102	16.6	97	15.7		86	15.8	11	15.1	
2	1136	17.1	120	19.4		99	18.1	21	28.8	
3+	665	10.0	75	12.1		57	10.4	18	24.7	
Severity of disability					<.01					.71
Mild	4367	65.7	318	51.4		282	51.6	36	49.3	
Severe	2275	34.3	301	48.6		264	48.4	37	50.7	
Type of disability					<.01					.47
Physical disability	2861	43.1	214	34.6		187	34.2	27	37.0	
Brain lesions	662	10.0	74	12.0		62	11.4	12	16.4	
Visual disturbance	661	10.0	40	6.5		35	6.4	5	6.8	
Hearing impairment	1192	17.9	103	16.6		90	16.5	13	17.8	
Other	1266	19.1	188	30.4		172	31.5	16	21.9	

Abbreviations: CCI, Charlson Comorbidity Index; COVID-19, coronavirus disease 2019.

 The COVID-19 infection rate did not statistically significantly differ according to gender (*P* = .12). No children with disabilities who were aged 0–9 years presented with COVID-19 infection; the COVID-19 infection rate was the highest in the 10–19 year age group. The COVID-19 infection rate was higher among Medical Aid recipients (16.0%) as compared with NHI members. The COVID-19 infection rate tended to increase with increasing CCI, but the trend was not statistically significant (*P* = .13). The COVID-19 infection rate was higher among those with severe disabilities (11.7%) as compared with those with mild disabilities (6.8%). The COVID-19 infection rate was the highest among those with “other” types of disabilities (12.9%), followed by those with brain lesions (10.1%), hearing disabilities (8.0%), physical disabilities (7.0%), and visual disabilities (5.7%). The COVID-19 mortality rate differed according to age and CCI among people with disabilities; mortality increased with increasing age and CCI.

###  Logistic Regression Analysis for COVID-19 Infection and Death

 The association between participant characteristics and economic status and the risk of COVID-19 infection were analyzed (Table 2).

**Table 2:** Odds Ratios for COVID-19 Infection in People With Disabilities

	**Univariate Model**			**Multivariate Model 1**			**Multivariate Model 2**		
	**Crude OR**	**95% CI**	***P *** **Value**	**Adjusted OR**	**95% CI**	***P *** **Value**	**Adjusted OR**	**95% CI**	***P *** **Value**
Gender									
Male	1 (Ref)	1 (Ref)	1 (Ref)	1 (Ref)	1 (Ref)	1 (Ref)	1 (Ref)	1 (Ref)	1 (Ref)
Female	0.88	0.74-1.03	.12	0.85	0.72-1.01	.06	0.84	0.71-1.00	.05
Age (y)									
0-29	1 (Ref)	1 (Ref)	1 (Ref)	1 (Ref)	1 (Ref)	1 (Ref)	1 (Ref)	1 (Ref)	1 (Ref)
30-39	1.44	0.80-2.58	.22	1.52	0.84-2.76	.17	1.59	0.88-2.89	.13
40-49	1.96	1.23-3.13	.01	1.97	1.22-3.18	.01	2.12	1.31-3.44	<.01
50-59	1.82	1.21-2.73	<.01	1.73	1.14-2.64	.01	1.86	1.21-2.85	.01
60-69	1.77	1.19-2.63	.01	1.89	1.25-2.86	<.01	1.99	1.31-3.04	<.01
70-79	1.30	0.87-1.96	.21	1.60	1.03-2.46	.04	1.68	1.08-2.63	.02
80+	1.15	0.76-1.74	.51	1.32	0.85-2.07	.22	1.37	0.86-2.18	.18
Health insurance premium									
Medical aid	1 (Ref)	1 (Ref)	1 (Ref)	1 (Ref)	1 (Ref)	1 (Ref)	1 (Ref)	1 (Ref)	1 (Ref)
1st quintile (lowest)	0.50	0.39-0.63	<.01	0.59	0.46-0.76	<.01	0.61	0.47-0.78	<.01
2nd quintile	0.30	0.21-0.43	<.01	0.35	0.24-0.50	<.01	0.35	0.24-0.51	<.01
3rd quintile	0.31	0.23-0.42	<.01	0.36	0.26-0.49	<.01	0.36	0.26-0.50	<.01
4th quintile	0.35	0.27-0.47	<.01	0.41	0.31-0.55	<.01	0.41	0.31-0.55	<.01
5th quintile (highest)	0.34	0.27-0.43	<.01	0.42	0.33-0.54	<.01	0.42	0.32-0.54	<.01
CCI score									
0	1 (Ref)	1 (Ref)	1 (Ref)	1 (Ref)	1 (Ref)	1 (Ref)	1 (Ref)	1 (Ref)	1 (Ref)
1	1.01	0.80-1.28	.96	1.01	0.79-1.28	.96	0.99	0.78-1.26	.93
2	1.21	0.97-1.50	.09	1.20	0.95-1.51	.12	1.18	0.94-1.49	.15
3+	1.29	0.99-1.68	.06	1.39	1.05-1.83	.02	1.35	1.02-1.78	.03
Severity of disability									
Mild	1 (Ref)	1 (Ref)	1 (Ref)	1 (Ref)	1 (Ref)	1 (Ref)	-	-	-
Severe	1.82	1.54-2.14	<.01	1.47	1.22-1.77	<.01	-	-	-
Type of disability									
Physical disability	1 (Ref)	1 (Ref)	1 (Ref)	-	-	-	1 (Ref)	1 (Ref)	1 (Ref)
Brain lesions	1.49	1.13-1.97	.01	-	-	-	1.39	1.05-1.85	.02
Visual disturbance	0.81	0.57-1.15	.23	-	-	-	0.81	0.57-1.15	.24
Hearing impairment	1.16	0.91-1.48	.25	-	-	-	1.26	0.97-1.63	.08
Other	1.99	1.61-2.44	<.01	-	-	-	1.63	1.29-2.06	<.01

Abbreviations: CCI, Charlson Comorbidity Index; COVID-19, coronavirus disease 2019; CI, confidence interval; OR, odds ratio.

 Multivariate Model 1 examined the association between health insurance premiums and disability severity and exposure to COVID-19 infection risk. We found that COVID-19 infection was not associated with gender (*P* = .12), but that the rate was higher in the 40-49 year (odds ratio [OR]=1.97, *P* = .01), 50-59 year (OR = 1.73, *P* = .01), 60-69 year (OR = 1.89, *P* < .01), and 70-79 year (OR = 1.60, *P* = .04) age groups as compared with the 0–29 year age group. Additionally, the exposure risk to COVID-19 infection was lower among NHI members as compared with Medical Aid recipients (*P* < .01) and was higher among those with a CCI of 3+ (OR = 1.39, *P* = .02) as compared with those with a score of 0. The COVID-19 infection risk was higher among those with severe disabilities (OR = 1.47, *P* < .01) as compared with those with mild disabilities.

 Multivariate Model 2 examined the association between health insurance premiums and disability type and COVID-19 infection risk. As in Model 1, COVID-19 infection was not associated with gender (*P* = .05), and the rate of infection was higher in the 40–49 year (OR = 2.12, *P* < .01), 50-59 year (OR = 1.86, *P* = .01), 60-69 year (OR = 1.99, *P* < .01), and 70-79 year (OR = 1.68, *P* = .02) age groups as compared with the 0–29 year age group. Additionally, the risk for COVID-19 infection was lower among NHI members as compared with Medical Aid recipients (*P* < .01) and higher among those with a CCI of 3+ (OR = 1.35, *P* = .03) as compared with those with a score of 0. The COVID-19 infection risk was statistically significantly higher among those with brain lesions (OR = 1.39, *P* = .02) and other types of disabilities (OR = 1.63, *P* < .01) as compared to those with intellectual disabilities.

 We analyzed the association between individual characteristics, including economic status, among people with disabilities and their COVID-19 mortality rate (among those with COVID-19 infection; Table 3).

**Table 3:** Odds ratios for COVID-19 Mortality in People With Disabilities

	**Univariate Model**			**Multivariate Model 1**			**Multivariate Model 2**		
	**Crude OR**	**95% CI**	***P*** ** Value**	**Adjusted OR**	**95% CI**	***P*** ** Value**	**Adjusted OR**	**95% CI**	***P*** ** Value**
Gender									
Male	1 (Ref)	1 (Ref)	1 (Ref)	1 (Ref)	1 (Ref)	1 (Ref)	1 (Ref)	1 (Ref)	1 (Ref)
Female	1.23	0.75-2.00	.41	0.89	0.51-1.53	.66	0.90	0.52-1.56	.71
Age (y)									
0-59	1 (Ref)	1 (Ref)	1 (Ref)	1 (Ref)	1 (Ref)	1 (Ref)	1 (Ref)	1 (Ref)	1 (Ref)
60-69	1.53	0.66-3.55	.33	1.72	0.73-4.05	.22	1.65	0.70-3.90	.26
70-79	2.66	1.17-6.05	.02	3.92	1.57-9.82	<.01	3.34	1.29-8.65	.01
80+	10.15	4.9-20.99	<.01	15.90	6.16-41.06	<.01	13.15	5.08-34.05	<.01
Health insurance premium									
Medical aid	1 (Ref)	1 (Ref)	1 (Ref)	1 (Ref)	1 (Ref)	1 (Ref)	1 (Ref)	1 (Ref)	1 (Ref)
1st quintile (lowest)	1.08	0.52-2.23	.84	0.66	0.29-1.50	.32	0.66	0.29-1.54	.34
2nd quintile	1.37	0.49-3.82	.55	0.96	0.30-3.10	.95	0.94	0.29-3.05	.92
3rd quintile	0.92	0.34-2.52	.87	0.87	0.29-2.55	.79	0.78	0.26-2.33	.66
4th quintile	0.94	0.39-2.27	.89	0.44	0.16-1.23	.12	0.43	0.16-1.17	.10
5th quintile (highest)	1.66	0.87-3.17	.13	0.75	0.35-1.64	.47	0.73	0.33-1.60	.43
CCI score									
0	1 (Ref)	1 (Ref)	1 (Ref)	1 (Ref)	1 (Ref)	1 (Ref)	1 (Ref)	1 (Ref)	1 (Ref)
1	1.69	0.79-3.61	.17	1.53	0.68-3.43	.30	1.44	0.64-3.23	.37
2	2.80	1.49-5.28	<.01	1.50	0.73-3.09	.28	1.52	0.73-3.15	.26
3+	4.17	2.12-8.23	<.01	1.40	0.61-3.21	.43	1.37	0.6-3.130	.45
Severity of disability									
Mild	1 (Ref)	1 (Ref)	1 (Ref)	1 (Ref)	1 (Ref)	1 (Ref)	-	-	-
Severe	1.10	0.67-1.79	.71	2.49	1.33-4.64	<.01	-	-	-
Type of disability									
Physical disability	1 (Ref)	1 (Ref)	1 (Ref)	-	-	-	1 (Ref)	1 (Ref)	1 (Ref)
Brain lesions	1.34	0.64-2.80	.44	-	-	-	1.51	0.65-3.51	.34
Visual disturbance	0.99	0.36-2.74	.98	-	-	-	0.94	0.30-2.93	.92
Hearing impairment	1.00	0.49-2.03	1.00	-	-	-	0.78	0.35-1.73	.55
Other	0.64	0.34-1.24	.19	-	-	-	1.50	0.64-3.54	.35

Abbreviations: CCI, Charlson Comorbidity Index; COVID-19, coronavirus disease 2019; CI, confidence interval; OR, odds ratio.

 Multivariate Model 1 examined the association between health insurance premiums and disability severity and COVID-19 mortality risk. Age and disability severity were identified as predictors for COVID-19 mortality among people with disabilities. COVID-19 mortality risk was higher in the 70–79 year (OR = 3.92, *P* < .01) and 80+ year (OR = 15.90, *P* < .01) age groups as compared with those aged 0–59 years and was likewise higher among individuals with severe disabilities (OR = 2.49, *P* < .01) as compared with those with mild disabilities. However, an interest variable in this model—health insurance premiums—was not a risk factor for COVID-19 mortality. Multivariate Model 2 (Table 3), a model regarding the association between health insurance premiums and disability type and COVID-19 mortality risk, showed age as the only predictor of COVID-19 mortality. COVID-19 mortality risk was higher in the 70–79 year (OR = 3.34, *P* = .01) and 80+ year (OR = 13.15, *P* < .01) age groups as compared with those aged 0–59 years, and the associated interest variables — health insurance premium and disability type — were not statistically significant predictors of COVID-19 mortality risk.

## Discussion

 The current study analyzed the association between the demographic and medical characteristics among people with disabilities and exposure to COVID-19 infection and death in Korea. The results showed that COVID-19 infection risk was higher among the lower income class, those with severe disabilities, and those with brain lesions or “other” types of disabilities (ie, speech disabilities, hepatic dysfunction, respiratory dysfunction, facial disfigurement, intestinal fistular/urinary disabilities, epilepsy, intellectual disabilities, autistic disorder, and mental disorders). COVID-19 mortality risk was 15.90 times higher in the 80+ year age group with COVID-19 infection as compared with younger groups and was 2.49 times higher among those with severe disabilities as compared with those with mild disabilities. Disability type did not predict mortality risk. In line with the higher COVID-19 infection rate reported among people with disabilities (despite the US Centers of Disease Control and Prevention report that disability itself is not a risk factor for COVID-19),^14^ our findings show that COVID-19 infection and mortality risks vary within the disabled population as well.

 Specifically, within the disabled population, we found that infection and mortality rates differed as follows. First, rates differed according to the severity of the disability. According to a British study analyzing the association of disability severity and COVID-19 risk, conducted between January 24, 2020 to November 20, 2020, the risk for COVID-19 mortality was 1.9 times higher among male patients with mild disabilities and 3.1 times higher among male patients with severe disabilities as compared to their non-disabled counterparts. Similarly, the risk for COVID-19 mortality was 2.0 times higher among female patients with mild disabilities and 3.5 times higher among female patients with severe disabilities as compared to their non-disabled counterparts. Although the overall mortality rate decreased after adjusting for individual and household characteristics, the mortality rate remained higher among those with disabilities as compared with the non-disabled population.^8^ People with severe disabilities are more readily exposed to the virus, as it is more difficult for them to access government guidelines and accurate information pertaining to the COVID-19 pandemic, rendering it challenging for them to take precautions against the infection or to take appropriate protective measures.^15^ Moreover, the prevalence of pre-existing conditions is higher among people with disabilities as compared to the non-disabled population,^16^ and COVID-19 infection can be more fatal among people with severe disabilities (especially those with poor mobility) if they have limited access to healthcare facilities due to social distancing and self-quarantine measures and consequently fail to receive timely treatment.^3,17^

 Second, COVID-19 infection and mortality rates differed according to income in the current study. A US study analyzing social inequities in COVID-19 infection rates reported that infection rates were higher among men, older adults (aged 65 and over), ethnic minorities (Blacks, Asians, Hispanics, Native Americans), and within the disabled population, where the infection rate was higher among people living in poverty as compared to those with a high income.^18^ Another study analyzing the association of socioeconomic inequity and COVID-19 risk in the US reported similar findings. According to the said study, the COVID-19 infection rate was higher in regions densely populated by a variety of ethnicities, and low education levels and income levels elevated the risk for COVID-19 infection.^19^ In the present study, income level was associated with infection rate; however, mortality was not associated with low income among people with disabilities. As income disparity is not a biological risk factor for infection, these findings suggest that mortality risk can be reduced through appropriate interventions.^20^

 Third, we found that the observed rates differed according to disability type. In the present study, people with “other” types of disabilities, including intellectual disabilities, were most vulnerable to infection; however, disability type was not statistically significantly associated with mortality rate. In contrast, previous studies mostly report that disability type is more strongly associated with COVID-19 mortality as compared with infection. A study analyzing associations between intellectual/developmental disabilities and COVID-19 risk in 547 US healthcare facilities found that intellectual and developmental disability is a potent predictor of COVID-19 infection and mortality, even after adjusting for other confounders.^21^ Additionally, a study conducted among people with physical and developmental disabilities and their non-disabled counterparts in New York reported that the COVID-19 fatality rate was higher among New York residents with physical and/or developmental disabilities (15%) as compared with the non-disabled population (7.9%), with a mortality rate of 1175 per 100 000 population among people with physical and developmental disorders and a mortality rate of 151 per 100 000 for people without disabilities.^9^

 In addition to the classification system that categorizes these disabilities into the same category as “other,” the differences in the results are derived from the fact that although people with intellectual disabilities in Korea reside in institutions (38.71% as of December 2019) and, therefore are more vulnerable to infection, a higher mortality is not seen in this population. In fact, the cumulative mortality rate in Korea is very low (5.848 per 100 000 population) compared to that in other countries; thus, despite the higher vulnerability, mortality is not affected.^1,22^

## Limitations and Conclusions

 This study has several limitations that we acknowledge here. First, while the number of daily new confirmed COVID-19 cases in Korea recently exceeded 10 000 (as of January 26, 2022),^23^ the data in this study covers the 5 months from January 2020 during the first epidemic in Korea. Therefore, the small size of data collected from this period could have affected the results. Second, the data present an inadequate classification of people with disabilities. As previously mentioned, disabilities are classified into 15 types in Korea; however, these were merged into five groups in the NHIS-COVID-19 DB. This classification system hinders a more detailed examination of characteristics and differences across different types of disabilities. Another study limitation pertains to the analysis of the causes of high infection and mortality rates among people with disabilities in the current investigation. Previous studies have confirmed that the COVID-19 infection and mortality rates are higher among people with disabilities as compared with their non-disabled counterparts; however, our study shed light on the differences in the factors associated with infection and mortality within the disabled population. The high infection and mortality rates observed among people with disabilities may be attributable to factors such as lack of family support and care (due to stricter social distancing measures) as well as poor infection control for institutionalized people with disabilities. However, these speculations cannot be confirmed definitively as there were limitations in establishing causal inferences and the direction of causality within the current investigation.

 Nevertheless, this study utilized a nationally representative NHIS-COVID-19 DB in order to compare gender, age, CCI, disability severity, and disability type in connection with COVID-19 infection and mortality rates among people with disabilities, thus presenting novel and innovative work within the resources of a rich epidemiologic dataset. In contrast to many previous studies comparing people with disabilities with their non-disabled counterparts, this study analyzed differences within the disabled population, demonstrating higher COVID-19 infection rates among those with a lower income, those with severe disabilities, and those with “other” disabilities (eg, intellectual as compared to physical disabilities). Health disparities among and within people with disabilities are evident not only within chronic disease but also within infectious disease, and the literature supports that this trend is similar with respect to COVID-19. Interventions targeting reductions in health disparities that affect people with disabilities are needed, in general and during infectious disease crises.

## Ethical issues

 This study conformed to the Korean Guidelines on De-identification of Personal Data and was approved by the Kyung Hee University Institutional Review Board [IRB No. KHSIRB-20-389 (EA)] as a review exemption study. As this study used de-identified data, informed consent was waived by the board. This research was conducted in accordance with the principles of the Declaration of Helsinki.

## Competing interests

 Authors declare that they have no competing interests.

## Authors’ contributions

 SYP conceived the study and designed it in collaboration with IHO. SYP and IHO contributed equally to this work. JYS performed data acquisition and statistical analysis. WHJ drafted the initial manuscript and performed the interpretation of the quantitative data. JNK provided critical revisions of the manuscript for important intellectual content. All authors reviewed and approved the final manuscript.

## Funding

 This work was supported by a grant of the Korea Health Technology R&D Project through the Korea Health Industry Development Institute (KHIDI), funded by the Ministry of Health & Welfare, Republic of Korea (grant number: HI21C2122).
